# Differential Effects of Chronic and Chronic-Intermittent Ethanol Treatment and Its Withdrawal on the Expression of miRNAs

**DOI:** 10.3390/brainsci3020744

**Published:** 2013-05-03

**Authors:** Gretchen van Steenwyk, Paulina Janeczek, Joanne M. Lewohl

**Affiliations:** Griffith Health Institute, School of Medical Sciences, Griffith University, Gold Coast Campus, Southport QLD 4215, Australia; E-Mails: gretchencvs16@gmail.com (G.S.); paulina.janeczek@griffithuni.edu.au (P.J.)

**Keywords:** alcoholism, neurotoxicity, gene expression

## Abstract

Chronic and excessive alcohol misuse results in changes in the expression of selected miRNAs and their mRNA targets in specific regions of the human brain. These expression changes likely underlie the cellular adaptations to long term alcohol misuse. In order to delineate the mechanism by which these expression changes occur, we have measured the expression of six miRNAs including miR-7, miR-153, miR-152, miR-15B, miR-203 and miR-144 in HEK293T, SH SY5Y and 1321 N1 cells following exposure to ethanol. These miRNAs are predicted to target key genes involved in the pathophysiology of alcoholism. Chronic and chronic-intermittent exposure to ethanol, and its removal, resulted in specific changes in miRNA expression in each cell line suggesting that different expression patterns can be elicited with different exposure paradigms and that the mechanism of ethanol’s effects is dependent on cell type. Specifically, chronic exposure to ethanol for five days followed by a five day withdrawal period resulted in up-regulation of several miRNAs in each of these cell lines similar to expression changes identified in post mortem human brain. Thus, this model can be used to elucidate the role of miRNAs in regulating gene expression changes that occur in response to ethanol exposure.

## 1. Introduction

Alcoholism and associated alcohol-use disorders are relatively common, with an estimated 76 million individuals world-wide drinking at levels considered to be high risk for short- and long-term harm [1]. Alcohol affects all organs of the body and is associated with an increased incidence of some types of cancers, greater susceptibility to inflammatory diseases, and difficulties with wound and bone healing [2]. Chronic alcohol misuse also results in persistent changes in brain function, which are manifested as tolerance, physical dependence, craving, and other behavioral changes [3]. 

It is now well established that these changes in brain function originate from alterations in gene expression that in turn underlie the cellular adaptations to chronic alcohol abuse [4,5]. Global gene expression studies have identified genes with altered expression following long-term alcohol consumption [6,7,8,9], as well as the effects of concomitant diseases such as liver cirrhosis [10]. These studies have identified genes that likely underlie the adaptive response of neurons in the prefrontal cortex, a brain region that is particularly susceptible to long-term alcohol abuse which include genes involved in protein trafficking, myelination, ubiquitination, apoptosis, cell adhesion, neurogenesis, and neural disease. The mechanism by which alcohol causes such diverse effects is not well understood. However, recent studies have identified a number of alcohol-responsive microRNAs (miRNAs), which are proposed to mediate these wide-ranging effects. 

MiRNAs are small, non-coding oligonucleotides ~22 nucleotides in length which predominantly target the 3' UTR of mRNA targets via strand complementarity. Due to their short sequence length, any one miRNA can affect hundreds of mRNA targets for either translation repression or RNA degradation; conversely, individual mRNA transcripts may be regulated by the co-ordinate action of several miRNAs [11]. Because miRNAs regulate many cellular functions, they may play significant roles in mediating the deleterious effects of ethanol in the brain. There is growing evidence that ethanol alters miRNA levels and miRNA-regulated systems that may determine effects such as ethanol-induced tolerance, gut leakiness, and neural stem cell proliferation and differentiation [12,13,14]. 

To date, the only study measuring the expression levels of miRNAs in alcoholic brain have been performed on the prefrontal cortex of uncomplicated alcoholics [15]. Many expression studies have been carried out using this brain region because it is particularly susceptible to the neurotoxic effects of alcohol misuse [16]. The study identified ~35 miRNAs, which are up-regulated in the prefrontal cortex of human alcoholics. Interestingly, the predicted target genes of the regulated miRNAs substantially overlap with genes known to be differentially expressed in the alcoholic prefrontal cortex. While studies using post mortem human brain have been informative, they are limited in their experimental design which each tissue sample representing a single time-point for a single individual. Alcohol-responsive miRNAs have also been identified by exposing cells in culture to well-established ethanol treatment paradigms [17]. This study found that chronic-intermittent exposure to ethanol and its withdrawal induced different patterns of miRNA expression in murine primary neuronal cultures [17] suggesting that mechanisms of miRNA-mediated gene regulation can be studied using *in vitro* models. 

We selected six miRNAs—miR-7, miR-152, miR-153, miR-144, miR-203 and miR-15B—which are predicted to target key genes involved in chronic alcoholism including GABA_A_ receptors [18], α-synuclein [19], regulators of G protein signaling [20], and the 14-3-3 family of molecular chaperones [21]. These miRNAs were selected on the basis of three criteria: The miRNAs were up-regulated in the prefrontal cortex of alcoholics compared with controls; the predicted targets of these miRNAs were significantly over-represented among genes down-regulated in the prefrontal cortex of the same individuals and; several of the miRNAs are predicted to target the same mRNA target. The expression of each of these miRNAs was measured in three human cell lines—HEK293T, SH SY5Y and 1321 N1 cells—following exposure to ethanol. These cells lines were selected to represent the most common cell lineages in brain. We chose HEK293T cells because they have many properties characteristic to immature neurons and express many neuronal genes. SH SY5Y cells are a dopaminergic neuroblastoma cell line commonly used in neuroscience research and 1321 N1 cells were selected for comparison since they are derived from an astrocytoma and therefore represent a completely different cell type. Comparisons have been made between two well-established treatment protocols with and without a withdrawal period to determine if these miRNAs are differentially expressed in response to ethanol in these cells. 

## 2. Results

We measured the changes in expression of six miRNAs (miR-7, miR-153, miR-152, miR-144, miR-203 and miR-15B) in HEK293T cells, SH SY5Y neuroblastoma and 1321 N1 cells following ethanol treatment. Each of these miRNAs was identified to be up-regulated in the prefrontal cortex of human chronic alcoholics [15]. Based on prior studies using cell culture models [17] and animal models of ethanol exposure [22,23] we compared miRNA expression levels following either a chronic or chronic-intermittent ethanol treatment paradigm. 

### 2.1. Effect of Alcohol Treatment on the Expression of miRNAs in HEK293T Cells

HEK293T cells expressed all six miRNAs under investigation although miR-144 and miR-203 were found at much lower levels than the other four miRNAs. Chronic exposure of these cells to 75 mM ethanol for five days resulted in a significant up-regulation of the expression of miR-7 and miR-144 and down-regulation of miR-203 and miR-15B with no significant change in the expression of miR-152 or miR-153 (Table 1). When cells were exposed to 75 mM ethanol for 5 days followed by a withdrawal period for 5 days, it again resulted in a distinct pattern of expression of these miRNAs. Interestingly, ethanol withdrawal resulted in an up-regulation of miR-7, miR-152, miR-203 and miR-15B similar to the expression changes seen in post mortem human brain. The expression of miR-144 was down-regulated to the extent that it could not be detected and the expression of miR-153 was unchanged.

**Table 1:** Fold Change in expression of miRNAs following chronic ethanol treatment and withdrawal.

	Chronic		Withdrawal	
	Fold Change	*P* Value	Fold Change	*P* Value
*HEK293T*				
miR-7	4.9	<0.0001	29.9	<0.0001
miR-15B	−1.7	0.023	3.2	<0.0001
miR-144	4.8	<0.0001	N/D	N/A
miR-152	1.8	N/S	9.0	<0.0001
miR-153	−1.5	N/S	1.1	N/S
miR-203	−1.7	0.037	3.8	<0.0001
*SHSY5Y*				
miR-7	−17.9	<0.0001	−14.7	<0.0001
miR-15B	−9.2	<0.0001	−3.0	0.002
miR-152	−1.7	0.024	1.4	N/S
miR-153	−1.7	NS	2.9	N/S
miR-203	N/D	N/A	N/D	N/A
*1321 N1*				
miR-7	2.0	N/S	28.1	<0.0001
miR-15B	−1.3	N/S	9.8	0.047
miR-152	1.0	N/S	6.2	<0.0001
miR-153	−2.7	0.013	7.0	0.043

The fold change in expression of each miRNA. Significance level is calculated in comparison to controls (Tukey HSD). Down-regulation is indicated by a negative fold change value. Chronic, 5 days treatment with 75 mM ethanol; Withdrawal, 5 days treatment with 75 mM ethanol followed by 5 days in ethanol free media. N/S, not significant; N/D, not detected, N/A, not applicable.

Chronic-intermittent exposure to 75 mM ethanol (12 h on, 12 h off) for 5 days followed by removal of ethanol for five days resulted in a significant up-regulation in the expression of miR-7 which returned to normal levels following ethanol removal. The expression of miR-203 was significantly reduced following chronic-intermittent ethanol exposure, and this reduced expression level persisted following the 5 day withdrawal period. The expression of miR-152, miR-144, miR-15B and miR-153 did not change following either chronic-intermittent ethanol exposure or its removal (Table 2).

### 2.2. Effect of Alcohol Treatment on the Expression of miRNAs in SH SY5Y Cells

SH SY5Y cells expressed all of the miRNAs except for miR-144. In addition, miR-203 was expressed at much lower levels than the other four miRNAs. Chronic exposure of these cells to 75 mM ethanol for five days resulted in a significant down-regulation of the expression of miR-7, miR-15B and miR-152 with no change in the expression of miR-153. The expression of miR-203 was down-regulated to the extent that it could not be detected. When cells were exposed to 75 mM ethanol for 5 days followed by a withdrawal period for 5 days, the expression of miR-7 and miR-15B was significantly down-regulated, the expression of miR-152 and miR-153 was unchanged and miR-203 was down-regulated to the extent that it could not be detected (Table 1).

**Table 2:** Fold Change in expression of miRNAs following chronic-intermittent ethanol treatment and withdrawal.

	Chronic-Intermittent		Withdrawal	
	Fold Change	*P* Value	Fold Change	*P* Value
*HEK293T*				
miR-7	2.9	0.018	1.4	N/S
miR-15B	1.0	N/S	−1.3	N/S
miR-144	1.6	N/S	1.4	N/S
miR-152	1.0	N/S	−1.5	N/S
miR-153	1.1	N/S	−1.3	N/S
miR-203	−2.0	0.012	−2.3	0.005
*SH SY5Y*				
miR-7	1.2	N/S	−1.7	0.017
miR-15B	1.5	N/S	1.0	N/S
miR-152	1.7	N/S	0.9	N/S
miR-153	7.3	<0.0001	1.5	N/S
miR-203	1.8	0.013	1.0	N/S
*1321 N1*				
miR-7	1.6	N/S	4.2	0.001
miR-15B	1.0	N/S	1.7	N/S
miR-152	1.1	N/S	1.6	N/S
miR-153	1.0	N/S	1.8	N/S

The fold change in expression of each miRNA. Significance level is calculated in comparison to controls (Tukey HSD). Down-regulation is indicated by a negative fold change value. Chronic-Intermittent, 5 days intermittent treatment with 75 mM ethanol; Withdrawal, 5 days intermittent treatment with 75 mM ethanol followed by 5 days in ethanol free media. N/S, not significant.

Chronic-intermittent exposure to 75 mM ethanol (12 h on, 12 h off) for 5 days followed by removal of ethanol for five days resulted in quite different patterns of expression for each miRNA. The expression of miR-7 was unchanged following chronic-intermittent treatment but decreased by ~2 fold following ethanol removal. The expression of miR-153 was up-regulated by ~7 fold following chronic-intermittent treatment and returned to near normal levels once ethanol was removed. The expression of miR-203 and miR-152 (~2 fold) was up-regulated following chronic-intermittent ethanol treatment and returned to near normal levels once ethanol was removed (Table 2).

### 2.3. Effect of Alcohol Treatment on the Expression of miRNAs in 1321 N1 Cells

The 1321 N1 astrocytoma-derived cell line expressed four out of the six miRNAs. MiR-7, miR-152, miR-153 and miR-15B were expressed in the 1321 N1 cell line whereas miR-144 and miR-203 were below the threshold for reliable detection. Chronic exposure of these cells to 75 mM ethanol for five days resulted in a significant down-regulation of the expression of miR-153 whereas the expression of miR-7, miR-152 and miR-15B was not significantly altered (Table 1). When these cells were exposed to 75 mM ethanol for 5 days followed by a withdrawal period for 5 days, it resulted in a distinct pattern of expression of these miRNAs. The expression of all four miRNAs was significantly up-regulated. Chronic-intermittent exposure to 75 mM ethanol (12 h on, 12 h off) for 5 days followed by removal of ethanol for five days did not change the expression of any of the miRNAs. Interestingly, miR-7 was up-regulated following the removal of ethanol with no change in the other three miRNAs (Table 2). 

### 2.4. Comparison between Cell Lines

**Figure 1:**
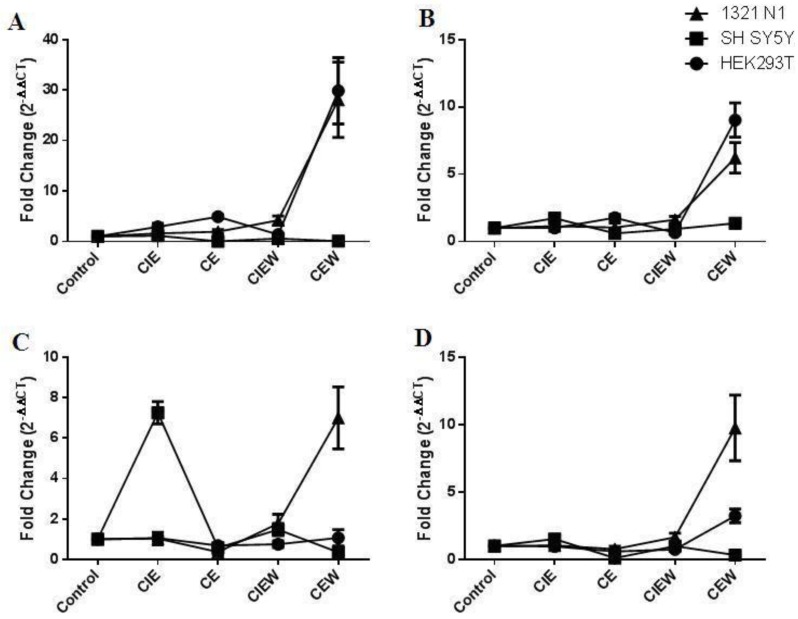
The pattern of expression of miRNAs following ethanol treatment. (**A**) miR-7; (**B**) miR-152; (**C**) miR-153 and (**D**) miR-15B. Data is plotted as mean ± SEM.

Four of the six miRNAs were expressed by all three cell lines under investigation. Each of these miRNAs shows a distinct pattern of expression following ethanol exposure and this pattern is dependent on the ethanol exposure paradigm used. Furthermore, the expression pattern of these four miRNAs was also significantly different between the three cell lines studied (MANOVA, Cell Line × Treatment Group, miR-7, *F*_2,8_ = 9.92 *P* < 0.0001; miR-153, *F*_2,8_ = 15.35 *P* < 0.0001; miR-152, *F*_2,8_ = 10.62 *P* < 0.0001; miR-15B, *F*_2,8_ = 14.58 *P* < 0.0001). The overall profile of expression across the different treatment groups is shown in Figure 1. The most significant differences in the effect of ethanol exposure on miRNA expression in each cell line is apparent following the chronic ethanol plus withdrawal (CEW) treatment paradigm. 

## 3. Discussion

Chronic and excessive alcohol misuse results in changes in miRNA expression and function that underpin the neurodegenerative effects of alcohol by changing the gene expression profile of key targets in susceptible neurons. The precise mechanism by which neuronal damage occurs is not completely understood, however recent evidence suggests that alcohol exposure results in changes in miRNAs which in turn alter gene expression profiles in susceptible brain regions [15]. Thus, miRNAs are likely to act as “master regulators” mediating cellular changes resulting from alcohol neurotoxicity. 

We measured the expression of six miRNAs—miR-7, miR-153, miR-152, miR-15B, miR-203 and miR-144—which are predicted to target key genes involved in chronic alcoholism and other neurodegenerative diseases. Chronic and chronic-intermittent exposure to ethanol, as well as its removal, resulted in specific changes in the expression of miRNAs in each cell line suggesting that different expression patterns can be elicited with different exposure paradigms and that the mechanism of ethanol’s effects is dependent on cell type.

Although HEK293T cells are derived from human embryonic kidney cells (HEK), they have many properties characteristic to immature neurons [24,25]. Additionally, transcript and protein expression profiles and miRNA analyses demonstrate that they express many neuronal genes and may in fact have a neuronal lineage [26]. They are also easy to culture and readily transfectable and are thus, ideally suited for use in studies designed to elucidate the miRNA regulation of neuronal gene expression. Chronic ethanol exposure resulted in a significant up-regulation of the expression of miR-7 and miR-144 and down-regulation of miR-203 and miR-15B. Interestingly, ethanol withdrawal resulted in an up-regulation of miR-7, miR-152, miR-203 and miR-15B similar to the expression changes seen in post mortem human brain [15]. 

SH SY5Y cells are a dopaminergic neuroblastoma cell line commonly used in neuroscience research. Overall, exposure of these cells to chronic ethanol resulted in significant down-regulation of miR-7, miR-15B, miR-152 and miR-203 which persisted even after removal of ethanol. Chronic-intermittent exposure resulted in up-regulation of miR-153 and miR-203. However, the levels of each of these miRNAs returned to near normal levels following ethanol removal. The expression of miR-7 was unchanged following ethanol exposure but decreased ~2-fold following ethanol removal.

The 1312 N1 cell line was originally derived from an astrocytoma and thus represents a completely different cell type to both HEK293T and SH SY5Y cells. This cell line was remarkably resistant to the effects of ethanol. Only one miRNA, miR-153 was down-regulated following chronic treatment and no miRNA showed changes in expression following chronic-intermittent treatment. However, chronic ethanol exposure followed by withdrawal resulted in a significant up-regulation of miR-7, miR-153, miR-152 and miR-15B. The same general trend was also seen following chronic-intermittent ethanol exposure and withdrawal, however, only miR-7 was significantly up-regulated.

One striking feature of the data presented was the differences in miRNA expression between cell lines following different ethanol exposure paradigms. Four of the six miRNAs were expressed in all three cell lines. Of particular note are the ways in which these cells respond to chronic ethanol exposure followed by a withdrawal period. All four miRNAs were up-regulated in 1321 N1 cells following chronic ethanol exposure plus withdrawal whereas only miR-7, miR-152 and miR-15B were up-regulated in HEK293T cells. Surprisingly, none of the miRNAs were up-regulated following this treatment paradigm in SH SY5Y cells suggesting that these cells have a different mechanism for miRNA regulation. This is further emphasized by the up-regulation of miR-153 following chronic-intermittent ethanol exposure which occurs only in SH SY5Y cells and not HEK293T or 1321 N1 cells. These findings emphasize the importance of cell type in studies measuring miRNA expression and function.

To date, only two other studies have reported changes in miRNA expression following exposure of cells to ethanol [13,17]. The most recent of these used primary cortical neuronal cultures and showed that exposure to ethanol results in selective changes in the expression of key miRNAs [17]. Two ethanol exposure paradigms were used; the first involved 10 days of chronic-intermittent exposure, where cells were exposed to 75 mM ethanol for 14 h followed by 10 h of ethanol-free media and the second involved 5 days of chronic-intermittent exposure followed by a 5 day withdrawal period. MiRNA expression was measured using an array based platform. As with the current study, each of these ethanol exposure paradigms resulted in distinct patterns of miRNA expression with 42 miRNAs differentially expressed following the chronic-intermittent treatment period and 26 miRNAs differentially expressed following the withdrawal period [17]. Only two of the six miRNAs measured in our study—miR-152 and miR-15B—were also differentially expressed in neuronal progenitor cells [17]. Similar to our findings, miR-152 was up-regulated by 2.74 fold following five days of chronic-intermittent ethanol exposure and five days of ethanol withdrawal but was not significantly up-regulated following ten days of chronic-intermittent exposure. MiR-15B was up-regulated by 2.3 fold following ten days of chronic-intermittent exposure and by 3.1 fold following five days of chronic-intermittent ethanol exposure and five days of ethanol withdrawal. 

The six miRNAs under investigation were selected on the basis of three criteria. The miRNAs were up-regulated in the prefrontal cortex of alcoholics compared with controls [15], the predicted targets of these miRNAs were significantly over-represented among genes down-regulated in the prefrontal cortex of the same individuals and several of the miRNAs are predicted to target the same mRNA target. Specifically, miR-203, miR-144, miR-15B and miR-153 are all predicted to target the α1 isoform of the GABA_A_ receptor, miR-7 and miR-153 are known to act co-operatively to regulate the expression of α-synuclein and miR-203, miR-144, miR-152, miR-7 and miR-15B are predicted to target isoforms of the 14-3-3 family. GABA_A_ receptor subunits [18], α-synuclein [19] and 14-3-3 isoforms [21] are all differentially expressed in the prefrontal cortex of human alcoholics. Since the function of very few miRNAs have been studied in detail, the identification of miRNA targets is based on bioinformatic prediction algorithms and direct experimental evidence of miRNA:mRNA interactions is required to determine if these mRNAs are regulated by these miRNAs in the brain.

Studies with post mortem alcoholic brain have identified ~35 miRNAs which are up-regulated by alcohol exposure [15] and these may be involved with the pathophysiology of alcoholic brain damage. Although studies utilizing post mortem brain have been successfully used to delineate gene expression profiles characteristic of uncomplicated and cirrhotic alcoholics compared with controls [6,7,8,9], they are limited by availability of tissue, and that each brain sample represents a single time point for a single individual. These tissues are not amenable to manipulation and cannot be used to investigate the mechanism by which gene expression changes occur in brain. Thus, although many differentially expressed mRNA transcripts and miRNAs have been identified, the mechanism by which gene expression changes occur remains elusive. 

Here, we have exposed three different cell lines to ethanol using two different treatment paradigms in order to develop a model system that can be used to investigate the role of miRNAs in modifying gene expression following ethanol exposure. The ethanol exposure paradigms chosen were based on well-established animal treatment models as well as previously published cell culture models [17,27,28]. Cells were exposed to ethanol (75 mM) for five days using either a chronic exposure paradigm, where cells are continuously exposed to ethanol, or a chronic-intermittent exposure paradigm, where cells are exposed to and withdrawn from ethanol (75 mM) in repeating 12 h cycles. Cells were harvested either directly following the ethanol treatment or after a five day withdrawal period in which ethanol was removed. While the ethanol exposure paradigms used here cannot be directly compared to the drinking patterns of human alcoholics, it is interesting to note that up-regulation of miRNAs was observed in these cell lines, particularly following ethanol withdrawal. Thus, this study highlights the strength of the developed ethanol-exposure cell culture model in measuring ethanol-responsive miRNA expression changes. Variations in expression patterns between chronic and chronic-intermittent exposure supports the ability to induce ethanol-responsive expression changes in a cell culture model, and show that the frequency and/or duration of exposure are the primary variables responsible for differences in expression. 

## 4. Experimental Section

### 4.1. Cell Culture

SH SY5Y, HEK293T and 1321 N1 cells were grown in Dulbecco’s Modified Eagle Medium; Type 11995 (DMEM; Life Technologies Australia Pty Ltd., Mulgrave, Vic, Australia) supplemented with 10% (v/v) foetal bovine serum (FBS; Life Technologies Australia Pty Ltd.) and treated with 50 U Penicillin/50 µg streptomycin (Pen/Strep; Life Technologies Australia Pty Ltd.) per mL media. Cells were incubated under 5% CO_2_ at 37 °C. At 80% confluence the cells were passaged, a 10 µL sample of the cells was counted using a haemocytometer. 

**Figure 2:**
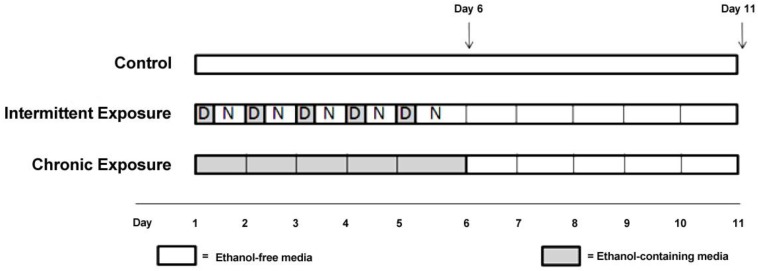
Experimental Design.

Approximately 600,000 cells per 50 cm^2^ flask were seeded into each of five treatment flasks (Figure 2), with 5 mL of 10% FBS (v/v) DMEM and incubated under normal conditions (37 °C and 5% CO_2_), until the cells were 80% confluent. At this point the media in each flask was replaced with media (DMEM) without FBS, to prevent cell proliferation during the treatment period. Cells were rinsed with Dulbecco’s Phosphate-Buffered Saline (DPBS) before each media change. For ethanol treatment experiments, flasks were assigned to one of five experimental groups, chronic (CE), chronic plus withdrawal (CWE), chronic-intermittent (CIE), chronic-intermittent plus withdrawal (CIWE) or control. CE cells were exposed to 75 mM ethanol continuously for 5 days and then harvested. CWE cells were exposed to 75 mM ethanol continuously for 5 days followed by a 5 day withdrawal period prior to harvest. CIE cells were exposed to 75 mM ethanol for 5 days in alternating cycles—12 h of ethanol exposure followed by 12 h of ethanol-free media. CIWE cells were exposed to 75 mM ethanol for 5 days of alternating cycles—12 h of ethanol exposure followed by 12 h of ethanol-free media followed by a 5 day withdrawal period prior to harvest. Control flasks were kept in ethanol-free media and subjected to media changes at the same time as ethanol exposed cells. Control flasks were harvested at both timepoints; *i.e.*, after 5 days and after 10 days (C5 and C10). The ethanol treated cells were maintained in a sealed vessel in which the atmosphere was saturated with ethanol in order to maintain the ethanol concentration at the level added to the medium. Ethanol concentration in the media was monitored every 24 h using an ethanol assay kit (Abcam, Sapphire Bioscience Pty. Ltd., Redfern, Australia).

The cells were harvested with 1 mL of QIAzol^®^ (Qiagen Pty Ltd., Chadstone Centre, Vic, Australia) in each flask and incubated for approximately 5 min at room temperature, until the cells had detached from the flask. The homogenised cell mixture was then transferred into individual 1.5 mL tubes and stored at −80°C. 

### 4.2. RNA Extraction

RNA was extracted from the cells with the miRNeasy Mini Kit and the RNeasy MinElute Kit (Qiagen). Extractions were carried out as per the manufacturer’s handbook. The RNA was extracted into two separate fractions; one fraction with all RNA greater than 200 nt and another miRNA-enriched fraction containing RNA less than 200 nt. RNA quantity was measured by absorbance at 260 nm using the NanoDrop^®^ 1000 Spectrophotometer (Thermo Scientific, Waltham, MA, USA). 

### 4.3. Reverse Transcription

The small RNA fractions were reverse transcribed with the miScript II RT Kit (Qiagen), to synthesise cDNA for each of the samples. Each reaction consisted of 1 µL template RNA, 1× miScript HiFlex Buffer, 1× Nucleics mix, 2 µL miScript Reverse Transcriptase mix, made up to a final volume of 20 µL with RNase-free water. The mix was incubated in a Mastercycler^®^ ep gradient S (Eppendorf, North Ryde, NSW, Australia) for 60 min at 37 °C, inactivated by heating to 95 °C for 5 min and then stored at −20 °C.

### 4.4. Real-Time PCR

Reactions were performed with the miScript SYBR^®^ Green PCR Kit (Qiagen). Samples were diluted to a 1/5 concentration and 1 µL of cDNA was used per reaction. Each sample was amplified in duplicate. MiScript Primer assays were also purchased from Qiagen. Each reaction consisted of 1× QuantiTect SYBR Green PCR Master Mix, 1× miScript Universal Primer Assay, 1× miScript Primer Assay, 1 µL template cDNA, in a final volume of 20 µL. Using a Rotor-Gene Q (Qiagen), the amplification cycle began with an initial activation of the DNA polymerase at 95 °C for 15 min, followed by 40 cycles of; 15 s at 94 °C, 30 s at 55 °C, 30 s at 70 °C. Tubes containing mastermix but no template were included for each primer set as negative controls. RNU6B was used as the internal reference for determining ΔC_T_ values. 

### 4.5. Statistical Analysis

Data was generated with Rotor-Gene Q series software (Qiagen). To determine the Cycle Threshold (C_T_) the threshold value was set at 0.1 in each reaction. The amplification plot of fluorescence *vs.* cycle number was used to set the threshold in the exponential phase of the reaction above the baseline. This was kept constant between runs to allow for analysis between experiments. The cycle threshold was calculated as the cycle number of an amplifying PCR product where it crosses the fixed threshold line. C_T_ values for each sample were normalized to the RNU6B reference snoRNA, and expressed as the ΔC_T_ value. In order to generate a fold-change comparison, ΔC_T_ values for controls (untreated cells) were subtracted from ΔC_T_ values for each treatment, generating a ΔΔC_T_ value. Note that the ΔCT of C5 cells were used to calculate ΔΔC_T_ values for the five day treatment paradigm and the ΔCT of C10 cells were used to calculate the ΔΔC_T_ values for the ten day treatment paradigm. Fold change values were calculated by converting ΔΔC_T_ values to 2^−ΔΔC^_T_ values [29].

The expression of each miRNA was compared between control flasks and each treatment group using Analysis of Variance (ANOVA), followed by a Tukey HSD post hoc test (SPSS version 19; IBM, Armonk, New York, NY, USA). Comparison of the profile of expression across treatments and between cell lines was performed using a Multivariate Analysis of Variance (MANOVA).

## 5. Conclusions

Chronic and excessive alcohol misuse results in changes in miRNA expression and function that underpin the neurodegenerative effects of alcohol by changing the gene expression profile of key targets in susceptible neurons. Here we measured the expression of six miRNAs—miR-7, miR-153, miR-152, miR-15B, miR-203 and miR-144—which are predicted to target key genes involved in chronic alcoholism and other neurodegenerative disease. Chronic and chronic-intermittent exposure to ethanol, as well as its removal, resulted in specific changes in the expression of miRNAs in each cell line suggesting that different expression patterns can be elicited with different exposure paradigms and that the mechanism of ethanol’s effects is dependent on cell type. Understanding the role of miRNAs in mediating the effects of alcohol exposure has important implications not only for understanding the pathophysiology of alcoholic brain damage, it also has the potential to identify new targets for therapeutic intervention. Thus, an important question is whether manipulating the expression or blocking the function of specific miRNAs can reverse the gene expression changes that occur as a result of alcohol exposure. 
